# A preliminary study of the miRNA restitution effect on CNV-induced miRNA downregulation in CAKUT

**DOI:** 10.1186/s12864-024-10121-8

**Published:** 2024-02-27

**Authors:** Kristina Mitrovic, Ivan Zivotic, Ivana Kolic, Jelena Zakula, Maja Zivkovic, Aleksandra Stankovic, Ivan Jovanovic

**Affiliations:** 1 Department of Radiobiology and Molecular Genetics, “Vinča” Institute of Nuclear Sciences, National Institute of the Republic of Serbia, University of Belgrade, 11001 Belgrade, P.O. Box 522, Serbia; 2 Department of Molecular Biology and Endocrinology, “Vinča” Institute of Nuclear Sciences, National Institute of the Republic of Serbia, University of Belgrade, 11001 Belgrade, P.O. Box 522, Serbia

**Keywords:** CAKUT, miRNA, CNV, miRNA mimic

## Abstract

**Background:**

The majority of CAKUT-associated CNVs overlap at least one miRNA gene, thus affecting the cellular levels of the corresponding miRNA. We aimed to investigate the potency of restitution of CNV-affected miRNA levels to remediate the dysregulated expression of target genes involved in kidney physiology and development in vitro.

**Methods:**

Heterozygous *MIR484* knockout HEK293 and homozygous *MIR185* knockout HEK293 cell lines were used as models depicting the deletion of the frequently affected miRNA genes by CAKUT-associated CNVs. After treatment with the corresponding miRNA mimics, the levels of the target genes have been compared to the non-targeting control treatment. For both investigated miRNAs, *MDM2* and *PKD1* were evaluated as common targets, while additional 3 genes were investigated as targets of each individual miRNA (*NOTCH3*, *FIS1* and *APAF1* as hsa-miR-484 targets and *RHOA*, *ATF6* and *CDC42* as hsa-miR-185-5p targets).

**Results:**

Restitution of the corresponding miRNA levels in both knockout cell lines has induced a change in the mRNA levels of certain candidate target genes, thus confirming the potential to alleviate the CNV effect on miRNA expression. Intriguingly, HEK293 WT treatment with investigated miRNA mimics has triggered a more pronounced effect, thus suggesting the importance of miRNA interplay in different genomic contexts.

**Conclusions:**

Dysregulation of multiple mRNA targets mediated by CNV-affected miRNAs could represent the underlying mechanism behind the unresolved CAKUT occurrence and phenotypic variability observed in CAKUT patients. Characterizing miRNAs located in CNVs and their potential to become molecular targets could eventually help in understanding and improving the management of CAKUT.

**Supplementary Information:**

The online version contains supplementary material available at 10.1186/s12864-024-10121-8.

## Background

Congenital anomalies of the kidney and urinary tract (CAKUT), a diverse group of urinary system malformations [1, 2] are the major cause of kidney failure in childhood [3]. Despite the evidence that CAKUT is a genetic disorder [4–6], the variable clinical outcome reflects the complex interaction between genetic, epigenetic, and environmental factors [7].

Over the last years, rare copy number variants (CNVs) (deletions and duplications ≥ 100 kb in size and frequency of less than 1:1,000 in population controls) have come up as potentially important molecular factors involved in CAKUT aetiology [6, 8–12]. It was previously suggested that nephrogenesis is very sensitive to variation in gene dosage, and the presence of kidney malformations should alert clinicians to the possibility of pathogenic genomic imbalances [6, 13]. Still, for a great proportion of CNVs identified in CAKUT, the researchers were unable to identify the precise causative protein-coding gene. Thus, further investigation of genetic elements beyond the protein-coding genes, located in CAKUT-associated CNVs is warranted. One of the possible genetic drivers of CAKUT could be miRNAs, the well-known regulators of gene expression [14]. Conditional knockout of Dicer, a microRNA-processing enzyme, in the developing renal tubules and parts of the ureteric bud in mice, results in CAKUT [15]. However, although the essential role of microRNA-dependent gene regulation in mammalian kidney development has been demonstrated, individual miRNAs located in rare CNVs in CAKUT have not been thoroughly investigated. It was shown previously that changes in miRNA gene dosage can affect the target gene’s expression in the corresponding individuals [16] and cell lines [10]. We previously mapped the miRNAs, taking into account the miRNA families, on CAKUT-associated rare CNV regions and performed bioinformatic analysis by which we identified the two most frequently affected miRNAs, hsa-miR-484 and hsa-miR-185-5p, in rare CNVs associated with CAKUT [10]. These miRNAs were selected for further analysis by meeting the criteria of being localized at one genomic locus and not having known interfering miRNAs with similar seed regions.

**Fig. 1:**
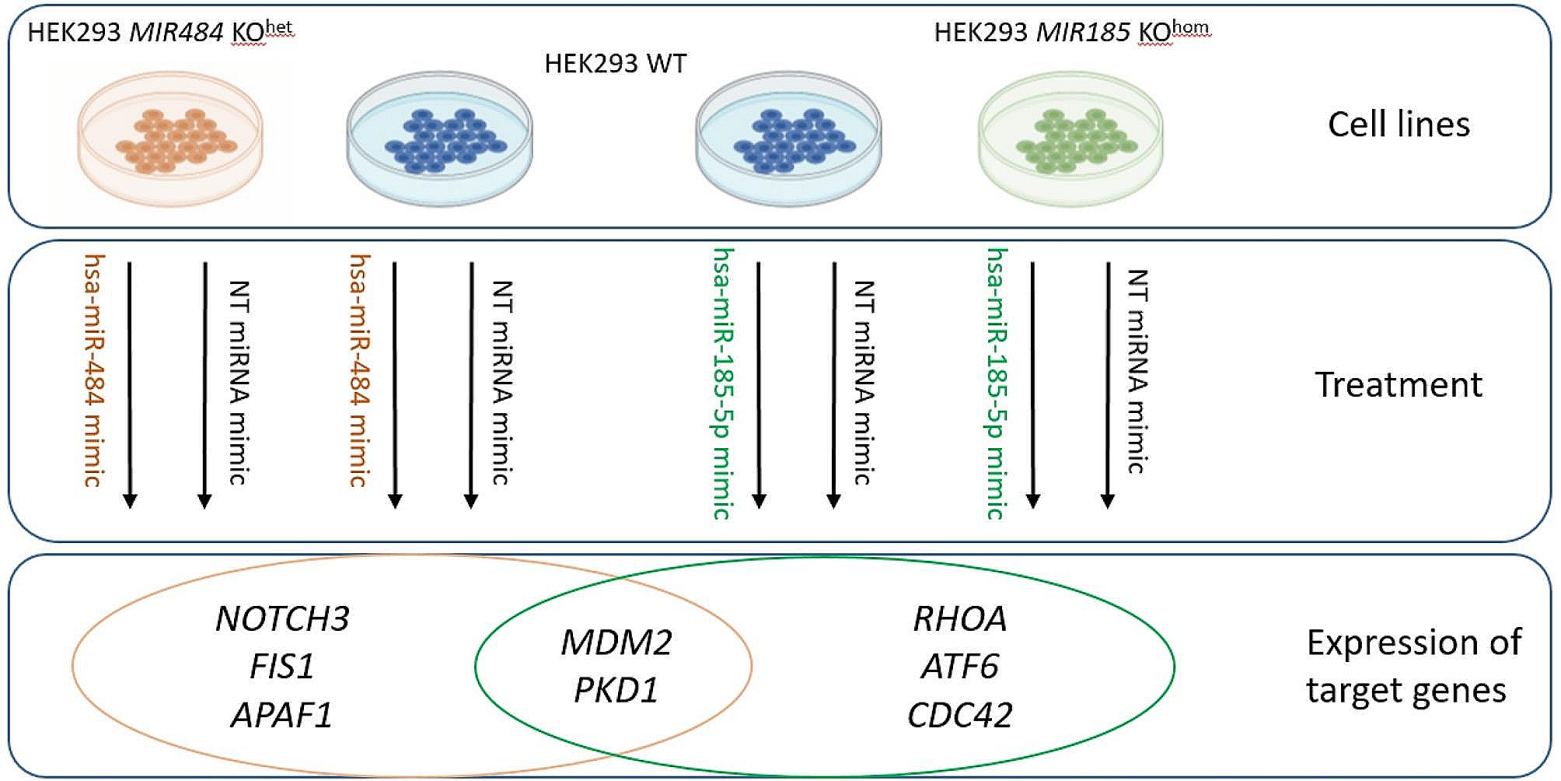
Study design. Heterozygous *MIR484* knockout HEK293 cell line (HEK293 MIR484 KO^het^) and homozygous *MIR185* knockout HEK293 cell line (HEK293 MIR185 KO^hom^) were used as models of CNV-affected miRNAs in CAKUT. Cell lines were treated simultaneously with the corresponding miRNA mimics and NT miRNA mimic. Two of the investigated genes (*MDMD2* and *PKD1*) have been selected as targets of both hsa-miR-484 and hsa-miR-185-5p mimics. Additional three candidate target genes with a potential role in CAKUT have been selected for each miRNA. As candidate target genes of hsa-miR-185-5p *RHOA, ATF6* and *CDC42* have been selected, while *NOTCH3, FIS1* and *APAF1* have been chosen as candidate target genes of hsa-miR-484. Both miRNA mimic treatments have been performed on HEK293 WT cell lines as well. Expression of target genes for miRNA mimic treatments have been compared to the corresponding NT miRNA treatments to explore the responsiveness of the expression of target genes to treatment with hsa-miR-484 and hsa-miR-185-5p mimics

Based on the before mentioned results, our aim was to employ the heterozygous MIR484 and homozygous MIR185 knockout HEK293 cell lines to investigate the CNV-induced dosage dependent miRNA downregulation and its molecular effect on target gene expression. The restitution of miRNA with corresponding mimics will be used to investigate the responsiveness of the dysregulated expression of target genes involved in kidney physiology and development in vitro (Fig. 1). These findings will represent a novel approach toward further untangling the genetic basis of CAKUT.

## Methods

### Cell lines

The in vitro model with the deletion of the frequently affected miRNA genes by rare CNVs identified in CAKUT (*MIR484* and *MIR185*) was designed. Human embryonic kidney cell line (HEK293 WT) and heterozygous *MIR484* (GeneID: 619553) knockout HEK293 cell line (HEK293 MIR484 KO^het^) were previously obtained for study Mitrovic et al., 2022 from Creative Biogene (Shirley, NY, USA), while homozygous *MIR185* (GeneID: 406961) knockout HEK293 cell line (HEK293 MIR185 KO^hom^) was additionally obtained from Creative Biogene (Shirley, NY, USA). Thus, two genomic scenarios have been covered. In the first scenario residual expression of the affected miRNA still exists while in the second scenario there is a complete knockout of the miRNA gene.

In brief, sgRNA was designed according to the *MIR484* and *MIR185* sequences, after which Cas9 and sgRNA vectors were co-transfected into HEK293 cells. The sgRNA sequences utilized for targeting *MIR484* were:

sgRNA1 (GGTTCCCAGCATGCCCCGCG) and sgRNA2 (ACTCCACAACGTGAAGCCAG), while the sgRNA sequences employed for targeting *MIR185* were: sgRNA1 (GCTTGGCTTAGGGAGCACAC), sgRNA2 (GCAAAGGCAAGGTCACAGGT) and sgRNA3 (CTGTGGCCTCGACGAAGACG) as reported by Creative Biogene (Shirley, NY, USA). To avoid off-target effects of CRISPR, the sgRNAs have been tested *in silico* taking into account the CRISPRs sensitivity to mismatches. Three days after transfection, pooled cells were collected to extract genomic DNA to confirm a successful knockout of *MIR484* and *MIR185*. The cell pool was serially diluted into 96-well plates in order for each cell to undergo monoclonal growth after which PCR and Sanger sequencing were done for further knockout validation. In this way each cell line originates from a single cell with identical genomic alterations, reducing genetic heterogeneity within the population. Positive HEK293 MIR484 KO^het^ clones, with a present wild type allele and 157 bp deletion allele and positive HEK293 MIR185 KO^hom^ clones, with 212 bp deletion alleles, were used for further analysis (Creative Biogene, Shirley, NY, USA).

### Cell culture and miRNA mimic treatment

Cell lines were maintained at 37˚C, 95% humidity and 5% CO2 in Dulbecco’s modified Eagle’s medium (DMEM) with 10% fetal bovine serum (FBS) and 1% penicillin/streptomycin. Cells were cultured in 25cm^2^ non-filter cap flasks. Before the transfection experiment, the cells were harvested to confirm the effect of the introduced knockout procedure on the corresponding miRNA genes expression.

After the establishment of cell cultures, in vitro transfection of the corresponding miRNA mimics was performed to evaluate the effect of the re-established level of affected miRNAs on the expression of the candidate target genes. One day before transfection, cells were seeded at a density of 1.5 × 10^5^ cells/well in a 6-well multiplate in antibiotics free media. Subsequently, 50nM of synthetic miRNA mimic was transfected into cells using Lipofectamine RNAiMax (Invitrogen, Carlsbad, CA USA) according to the manufacturer’s protocol. After 24 h, the medium with the transfection reagent was replaced with fresh DMEM medium. The cells were harvested 24 h after medium replacement (total of 48 h).

The synthetic miRNA mimics were employed in the following settings:

- hsa-miR-185-5p mimic (mirVana™ miRNA Mimic; Thermo Fisher Scientific Inc., Waltham, USA) was transfected into HEK293 MIR185 KO^hom^ and HEK293 WT.
- hsa-miR-484 mimic (mirVana™ miRNA Mimic; Thermo Fisher Scientific Inc., Waltham, USA) was transfected into HEK293 MIR484 KO^het^ and HEK293 WT.
- Non-targeting miRNA (NT miRNA), (mirVana™ miRNA Mimic; Thermo Fisher Scientific Inc., Waltham, USA) which does not produce identifiable cellular effects, was transfected in all investigated cell lines as control treatment.
- *GAPDH-*targeting mimic, (mirVana™ miRNA Mimic, Positive Control #2; Thermo Fisher Scientific Inc., Waltham, USA) was transfected in all investigated cell lines to serve as a positive control to confirm the successful transfection procedure.

All experiments have been performed in triplicate.

### Extraction of the total RNA from transfected cells

Prior to total RNA extraction, the medium was removed and cells were washed with 2 ml of cold PBS. Total RNA extraction was performed using TRI Reagent® solution (Ambion) according to the manufacturer’s protocol. Total RNA concentration and purity were determined with a NanoDrop ND-1000 spectrophotometer (Thermo Fisher Scientific Inc., Waltham, USA). RNA samples were dissolved in the nuclease free water (Ambion) and stored at -80 °C.

### Relative miRNA expression by quantitative real‑time PCR

To investigate the effect of the introduced knockout procedure hsa-miR-185-5p miRNA levels have been investigated in HEK293 MIR185 KO^hom^ cell lines. cDNA was generated from the total RNA using the TaqMan® microRNA reverse transcription kit according to manufacturer’s protocol (Thermo Fisher Scientific Inc., Waltham, USA). Primer pool for reverse transcription consisted of TaqMan® MicroRNA Assay reverse transcription primers for hsa-miR-185-5p (ID 002271) and RNU44 (ID 001094) at a final dilution of 0.05x. Synthesized cDNA was stored at -20 °C. The miRNA levels were quantified by quantitative Real-time PCR on the Applied Biosystems Real-Time 7500 system (Applied Biosystems, Inc., Foster City, CA). Stably expressed reference RNU44 was used as an endogenous control to normalize the hsa-miR-185-5p levels. All reactions were performed in duplicates in a 96-optical well plate at: 95 °C/10 min (1 cycle); 95 °C/15s, 60 °C/1 min (40 cycles).

### Selection of the target mRNA for the estimation of miRNA mimic treatment effect

Network analysis performed on miRNAs most frequently affected by rare CNVs, coupled with the functional enrichment analysis and literature research has been employed for the prioritization of the target genes associated with CAKUT [10]. The network algorithm has included only miRNAs known to be active in kidney tissue and miRTarBase v8.0 as a resource of validated miRNA-gene interactions [10]. Target genes of hsa-miR-484 and hsa-miR-185-5p, involved in the highest number of significantly enriched pathways (Cell cycle, Pathways in cancer, Neurotrophin signalling pathway) were further prioritized according to network topology and literature research. Regarding the network topology, we have investigated candidate genes which are targets of both hsa-miR-484 and hsa-miR-185-5p. Of the commonly targeted genes, CDC42 and MDM2 were involved in the largest number of previously described enriched pathways. Further literature investigation has confirmed the role of CDC42 in Slit-Robo pathway [17], associated with CAKUT [18], while novel data confirms that dysregulation of integrin-parvin-RAC1/CDC42 signaling leads to CAKUT [19]. In case of MDM2, its upregulation was observed in the tubulointerstitium of patients with tubulointerstitial fibrosis and in mice with unilateral ureteral obstruction (UUO) [20]. Additionally, MDM2 has pivotal role in cell cycle surveillance and it is involved in pathophysiological processes such as inflammation and fibrosis [20, 21], both crutial in chronic kidney disease. *RHOA* was the third gene selected from the network. Although it was regulated only by hsa-miR-185-5p, *RHOA* was involved in multiple described pathways including neurotrophin signaling pathway where it plays a crucial role in signal transmission important in early nephrogenesis [22]. Additionally, by investigating if the network harbors previously associated, highly penetrant CAKUT genes, we have identified only *PKD1*, being a target of both miRNAs.

Additional four candidate target genes (*APAF1*, *FIS1*, *ATF6* and *NOTCH3*) have been selected through literature research as candidate genes involved in CAKUT-related processes beyond the significantly enriched pathways identified in the network analysis.

Apoptosis has an important role in the development of the kidney and urinary tract [23] while the dysregulation of the apoptosis has been observed in certain CAKUT phenotypes [24]. Both, mitochondria and the endoplasmic reticulum (ER) significantly contribute to initiation of apoptosis [25, 26] thus both mechanisms have been covered with the representative genes. Dysregulation in the expression of genes involved in apoptotic processes can lead to the entire phenotypic spectrum of CAKUT during early development. Thus, *APAF1* was selected due to its central role in the apoptosis pathway [27]. Mitochondrial cell death pathways mediate apoptosis in response to endoplasmic reticulum (ER) stress. The interplay between the ER and mitochondria indicates the pivotal role of their interaction in controlling apoptosis [28]. FIS1, a protein controlling mitochondrial fission, is also involved in apoptosis [29, 30]. FIS1 interacts with BAP31, an ER membrane protein, leading to the activation of the procaspase-8, thus initiating and promoting apoptosis. This complex does not only connect these two organelles, but also transmits apoptotic signals from ER to mitochondria [31]. ATF6, selected due to its connection to ER stress and kidney dysfunction [32, 33], stands as a one of key components among the three branches activated during ER stress. Under normal conditions, ATF6 is bound to the ER and maintained in an inactive state. When ER stress develops, it triggers activation of the ATF6 which regulate the expression of CHOP, a central mediator of ER stress-induced apoptosis [34].

Notch signaling has a significant role in nephrogenesis [35–37] and accumulating evidence points to the involvement of Notch3 in various kidney diseases [38–40]. It functions as one of the major pathways that determine cellular identity during development, and has a function in cellular communication [41].

### Relative expression of the hsa-miR-484 and hsa-mir-185-5p target genes by quantitative real‑time PCR

cDNA was prepared using RevertAid First strand cDNA synthesis kit according to the manufacturer’s protocol (Thermo Fisher Scientific Inc., Waltham, USA) using 1 µg of total RNA. The mRNA levels of selected target genes in transfected cells were determined by quantitative Real-time PCR on ABI 7500 Fast RealTime PCR System (Applied Biosystems, Inc., Foster City, CA; Thermo Fisher Scientific Inc., Waltham, USA) using TaqMan® gene expression assays: Hs00540450_s1 for *MDM2* (Mouse double minute 2 homolog), Hs00947377_m1 for *PKD1* (Polycystin-1), Hs01128537_m1 for *NOTCH3* (Neurogenic locus notch homolog), Hs00211420_m1 for *FIS1* (Mitochondrial Fission 1 Protein), Hs00559441_m1 for *APAF1* (Apoptosis protease-activating factor-1), Hs00357608_m1 for *RHOA* (Ras homolog family member A), Hs00232586_m1 for *ATF6* (activating transcription factor 6), Hs00918044_g1 for *CDC42* (Cell division cycle 42), and Hs02786624_g1 for *GAPDH* (Glyceraldehyde 3-phosphate dehydrogenase). Relative mRNA levels were normalized using stably expressed reference gene, *GAPDH*. All reactions were performed in duplicates in a 96-optical well plate under the following conditions: 50 °C /2 min (1 cycle); 95 °C/10 min (1 cycle); 95 °C/15s, 60 °C/1 min (40 cycles).

### Statistical analysis of relative miRNA and mRNA levels

The relative levels of mature miRNAs and their target genes were calculated using the comparative Ct method [42]. The analysis of difference in relative mRNA and miRNA levels between groups was done using Student’s ttest. Values of *P* < 0.05 were statistically significant. All statistical analyses and graphical presentation of the results were performed using Prism v8 software (GraphPad Software, Inc.).

## Results

### Relative hsa-miR-185-5p levels and target gene mRNA levels in HEK293 MIR185 KO^hom^and HEK293 WT cell lines

**Fig. 2:**
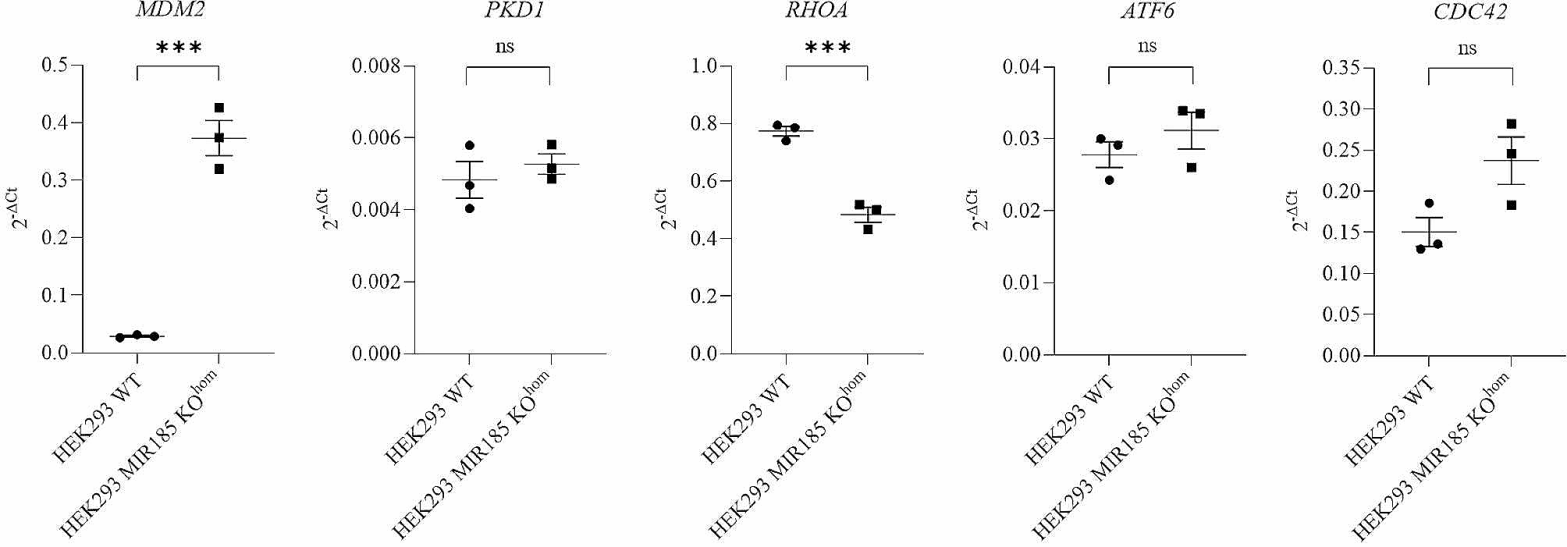
Difference in relative mRNA levels of hsa-miR-185-5p target genes between HEK293 MIR185 KO^hom^ and HEK293 WT cells. Relative mRNA levels were standardized against *GAPDH* endogenous control and presented as scatter plot of 2^− dCt^ values with standard errors of mean from three independent replicates. Student’s t-test, P_*MDM2*_=0.0004, P_*PKD1*_=0.494, P_*RHOA*_=0.0008, P_*ATF6*_=0.344 and P_*CDC42*_=0.063. *** denotes a significant difference at *P* < 0.001, ns– non significant

Initially, we have confirmed that hsa-miR-185-5p was not expressed in HEK293 MIR185 KO^hom^ cells (Additional File, Figure A1). A significant difference in mRNA expression between the HEK293 MIR185 KO^hom^ and HEK293 WT cell lines was observed for the genes *MDM2* and *RHOA*. The *MDM2* mRNA level was 12.77 fold increased while the *RHOA* mRNA level was 0.62 fold decreased in HEK293 MIR185 KO^hom^ compared to HEK293 WT cell lines (Student`s t-test, P_*MDM2*_=0.0004 and P_*RHOA*_=0.0008) (Fig. 2). On the other hand, *PKD1, ATF6* and *CDC42* did not show a significant difference in mRNA expression levels in HEK293 MIR185 KO^hom^ compared to HEK293 WT (Student`s t-test, P_*ATF6*_=0.344, P_*CDC42*_=0.063 and P_*PKD1*_=0.494, respectively) (Fig. 2). The downregulation of hsa-miR-484 and its association with dysregulation of target genes has been previously confirmed in HEK293 MIR484 KO^het^ cells [10].

### Relative mRNA levels of hsa-miR-185-5p and hsa-miR-484 target genes after transfection of miRNA mimics into the corresponding cell lines

The successful transfection procedure was confirmed by a substantial increase in the *GAPDH* Ct values after the transfection of *GAPDH-*targeting mimic into the cell lines thus confirming the downregulation activity. The successful transfection of hsa-miR-185-5p mimic into HEK293 MIR185 KO^hom^ (Additional File, Table A1) and HEK293 WT (Additional File, Table A2) cell lines, as well as the transfection of hsa-miR-484 mimic into HEK293 MIR484 KO^het^(Additional File, Table A3) and HEK293 WT (Additional File, Table A4) cell lines was confirmed.

**Fig. 3:**
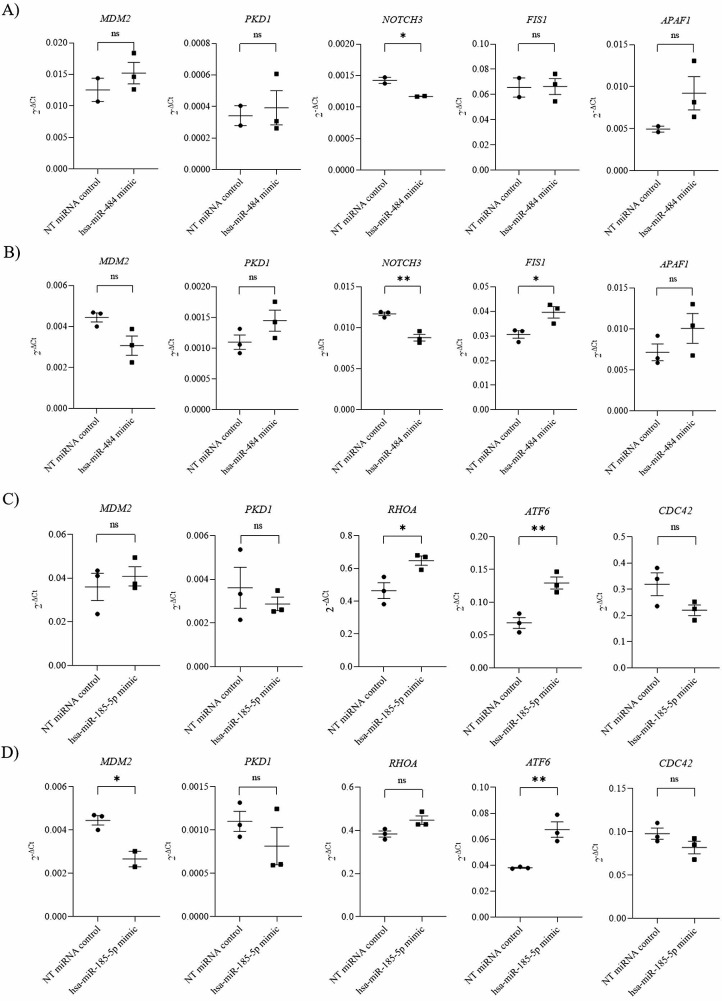
Difference in relative mRNA levels of hsa-miR-484 and hsa-miR-185-5p target genes after treatment of the cell lines with corresponding miRNA mimics. Relative mRNA levels were standardized against *GAPDH* endogenous control and presented as scatter plot of 2^− dCt^ values with standard errors of mean from independent replicates. (**A**) Relative mRNA levels of hsa-miR-484 target genes in HEK293 MIR484 KO^het^ after miRNA mimic treatment (**B**) Relative mRNA levels of hsa-miR-484 target genes in HEK293 WT after miRNA mimic treatment (**C**) Relative mRNA levels of hsa-miR-185-5p target genes in HEK293 MIR185 KO^hom^ after miRNA mimic treatment (**D**) Relative mRNA levels of hsa-miR-185-5p target genes in HEK293 WT after miRNA mimic treatment, Student’s t-test, * denotes a significant difference at *P* < 0.05; ** denotes a significant difference at *p* < 0.01, ns– non significant

A 0.82 fold decrease in *NOTCH3* mRNA level was observed in hsa-miR-484 mimic treated HEK293 MIR484 KO^het^ cells compared to NT miRNA treated HEK293 MIR484 KO^het^ cells (Student`s t-test, P_*NOTCH3*_=0.035) (Fig. 3; Table 1). The difference in expression levels of *MDM2*, *PKD1, FIS1* and *APAF1* in hsa-miR-484 mimic treated HEK293 MIR484 KO^het^ compared to NT miRNA treated HEK293 MIR484 KO^het^cells was not statistically significant (Student`s t-test, P_*MDM2*_=0.377, P_*PKD1*_=0.759, P_*FIS1*_=0.940 and P_*APAF1*_=0.198 respectively) (Fig. 3; Table 1).

**Table 1:** Expression of hsa-miR-484 and hsa-miR-185-5p target genes after treatment of the cell lines with corresponding miRNA mimics

Genes	Effect of		Effect of		Effect of		Effect of	
	hsa-miR-484 mimic on		hsa-miR-185-5p mimic on		hsa-miR-484 mimic on		hsa-miR-185-5p mimic on	
	HEK293 MIR484 KO^het^		HEK293 MIR185 KO^hom^		HEK293 WT		HEK293 WT	
	cell line		cell line		cell line		cell line	
	FC	P^**a**^**value**	FC	P^a^ value	FC	P^a^ value	FC	P^a^ value
*MDM2*	1.21	0.377	1.13	0.563	0.69	0.057	**0.60**	**0.019**
*PKD1*	1.14	0.759	0.79	0.496	1.31	0.164	0.74	0.306
*NOTCH3*	**0.82**	**0.035**	N/A	N/A	**0.75**	**0.003**	N/A	N/A
*FIS1*	1.01	0.940	N/A	N/A	**1.29**	**0.032**	N/A	N/A
*APAF1*	1.86	0.198	N/A	N/A	1.4	0.235	N/A	N/A
*RHOA*	N/A	N/A	**1.39**	**0.03**	N/A	N/A	1.16	0.055
*ATF6*	N/A	N/A	**1.88**	**0.007**	N/A	N/A	**1.77**	**0.008**
*CDC42*	N/A	N/A	0.69	0.107	N/A	N/A	0.83	0.169

Data marked in bold represents statistically significant differences in mRNA expression between miRNA mimic treated cells and NT mimic treated cells. FC–fold change in mRNA level after miRNA mimic treatment compared to NT mimic treatment; ^a^Student’s ttest

^a^Student’s ttest

An increase in mRNA level of 1.39 fold for *RHOA* and 1.88 fold for *ATF6* was detected in hsa-miR-185-5p mimic treated HEK293 MIR185 KO^hom^ cells compared to NT miRNA treated HEK293 MIR185 KO^hom^ cells (Student`s t test P_*RHOA*_=0.03, P_*ATF6*_=0.007) (Fig. 3; Table 1). The difference in expression levels of *MDM2, PKD1* and *CDC42* in hsa-miR-185-5p mimic treated HEK293 MIR185 KO^hom^ compared to NT miRNA treated HEK293 MIR185 KO^hom^ cells was not statistically significant (Student`s t-test, P_*MDM2*_=0.563, P_*PKD1*_=0.496 and P_*CDC42*_=0.107) (Fig. 3; Table 1).

The treatment of HEK293 WT has been performed separately with both miRNA mimics. A 0.75 fold decrease in *NOTCH3* mRNA level and 1.29 fold increase in *FIS1* mRNA level were observed in hsa-miR-484 mimic treated HEK293 WT cells compared to NT miRNA treated HEK293 WT cells (Student`s t-test, P_*NOTCH3*_=0.003 and P_*FIS1*_=0.032) (Fig. 3; Table 1). The difference in expression levels of *MDM2*, *PKD1* and *APAF1* in hsa-miR-484 mimic treated HEK293 WT cells compared to NT miRNA treated HEK293 WT cells was not statistically significant (Student`s t test, P_*MDM2*_=0.057, P_*PKD1*_=0.164, and P_*APAF1*_=0.235) (Fig. 3; Table 1).

A 0.60 fold decrease in *MDM2* mRNA level and a 1.77 fold increase in *ATF6* mRNA level were observed in hsa-miR-185-5p mimic treated HEK293 WT cells compared to NT miRNA treated HEK293 WT cells (Student`s t- test, P_*MDM2*_=0.019 and P_*ATF6*_= 0.008) (Fig. 3; Table 1). The difference in expression levels of *PKD1, RHOA* and *CDC42* in hsa-miR-185-5p mimic treated HEK293 WT cells compared to NT miRNA treated HEK293 WT cells was not statistically significant (Student`s t-test, P_*PKD1*_=0.306, P_*RHOA*_=0.055 and P_*CDC42*_=0.169) (Fig. 3; Table 1).

## Discussion

In the present study we have examined the translational potential of miRNAs affected by rare CNVs in CAKUT by performing in vitro miRNA mimic treatment on the two cell models which allow the comprehensive investigation of the CNV genomic defects observed in CAKUT patients. Hereby, we report the responsiveness of the dysregulated expression of target genes to treatment with hsa-miR-484 and hsa-miR-185-5p mimics.

In order to investigate the miRNA mimic treatment effect, we have employed two HEK293 cell models depicting heterozygous deletion of the *MIR484* gene and a complete knockout of the *MIR185* gene. Thus, we have covered two genomic scenarios where in the first scenario residual expression of the affected miRNA still exists while in the second scenario there is a complete knockout of the miRNA gene. Cells could maintain homeostasis through functional buffering and decrease or nullify the knockout effect. The complete knockout of the *MIR185* gene was introduced to achieve a more pronounced effect of miRNA gene loss and potentially stronger subsequent effect on target genes.

In the investigated settings, the most responsive effect was observed for the hsa-miR-185-5p/*RHOA* axis. While the knockout of the *MIR185* gene was associated with the downregulation of *RHOA* mRNA expression, the treatment of the HEK MIR185 KO^hom^ cell line using the hsa-miR-185-5p mimic resulted in the restitution of *RHOA* expression. Previously, it was described that *RHOA* knockdown (the effect observed in HEK MIR185 KO^hom^ cell line) in podocytes led to increased apoptosis [43], a process commonly associated with the development of kidney diseases [44]. The RHO family has a central role in cellular processes, including proliferation, differentiation and cell migration which are of essential importance in embryogenesis [45, 46]. Research on mice lacking the *RHO GDIα* gene, a regulator of RHO family members, showed that impairment of control of RHO family members induces altered kidney development [47]. Moreover, RHOA has been found to be present in the kidneys of rats and humans from the very early stages of renal development when most of the induction processes that characterize kidney organogenesis occur [45]. However, it should be noted that the observed effect does not depict the canonical activity of inhibitory miRNA gene expression regulation. Although, it is not uncommon that a miRNA can also act in-trans by upregulating target gene expression depending on tissue type and state with distinct context of transcripts and proteins [48]. For example, *MIR145* mediates myocardin gene upregulation during muscle differentiation [49], while the same miRNA induces ROCK1 downregulation in osteosarcoma [50]. Additionally, it was previously demonstrated that miR-185 inhibitor significantly up-regulated DNMT1 expression in HK2 cell line, and by modulating DNMT1 thereby regulating MEG3 expression in TGF-β1-induced renal fibrosis [51]. In our experimental settings, where miR-185 is knocked out in HEK293 cell line, DNMT1 could also be up-regulated and subsequently repress certain genes such as *RHOA*. Thus, future studies should follow to investigate the effect of hsa-miR-185-5p restitution on RHOA protein level to further untangle the effect of hsa-miR-185-5p on RHOA activity.

In case of *ATF6*, the upregulation in HEK293 MIR185 KO^hom^ compared to HEK293 WT cells was expected [32]. However, we have detected only a trend in elevation of the *ATF6* expression level. The treatment with corresponding hsa-miR-185-5p miRNA mimic of both, HEK293 MIR185 KO^hom^ and HEK293 WT cell lines triggered a significant increase in *ATF6* mRNA level, which was unexpected. A bioinformatic target prediction showed that the putative binding site for miR-185-5p was present in the 3′UTR of *ATF6*, and previously in TGF/beta1 exposed HK2 cells downregulation of *ATF6* mediated by miR-185-5p was confirmed [32]. However, for further investigation of renal cell embryonic development or differentiation an improved model system with controllable hsa-miR-185-5p coordinated expression pattern and consequently, influence on the expression of target mRNAs is warranted. This may include primary cell lines or kidney organoids offering new perspectives for the discovery of potentially novel pathways that are involved in disease pathogenesis and new drugs for future precision medicine strategies [52]. If the observed discrepancies occur due to the difference in cell lines employed in the studies, treatment difference (TGF beta treatment, and fibrosis) or knockout experiments, remains to be validated in future.

*MDM2* is a target gene for both investigated CNV miRNAs, which was shown to be upregulated in both, HEK293 MIR484 KO^het^ [10] and HEK MIR185 KO^hom^ cell lines compared to HEK293WT. However, the treatment with the miRNA mimics of the corresponding knockout cells did not trigger a significant effect on the *MDM2* transcript levels compared to the NT miRNA control. Contrary, treatment of HEK293 WT with hsa-miR-185-5p mimics was associated with the significant downregulation of the *MDM2* mRNA levels compared to NT miRNA control treatment while in case of hsa-miR-484 mimic treatment, a trend in downregulation of the *MDM2* mRNA levels compared to NT miRNA control treatment was observed. It should not be ruled out that certain changes in MDM2 levels could be the effect of p53 status of HEK293 cells. However, it was observed that the oversaturation of p53 in HEK293 cells prevents the notable change in activity of MDM2 even during the transfection of p53 [53]. Thus, even if changes in p53 levels occur it is not expected that the observed prominent changes in the expression of MDM2 could be triggered by the dysregulated p53.

One of the plausible mechanisms which could explain such discrepancies lies under the competing endogenous RNA (ceRNA) hypothesis. According to the hypothesis, a pool of non-coding RNAs holding miRNA response elements (MREs) could greatly influence miRNA activity by acting as a decoy [54]. Exogenously expressed miRNA decoys are long known to be able to inhibit miRNA function effectively and specifically [55–57]. Growing evidence describes the endogenous ceRNA decoys as competitively regulating the distribution of miRNA molecules onto their target genes for both hsa-miR-484 [58–60] and hsa-miR-185-5p [61–63]. Taking into account the previously described mechanism, the observed effects of more notable activity of transfected miRNA mimics in WT cell lines could be due to the more saturated decoys which allow further interactions of transfected mimics with their target transcripts. Contrary, the knockout system which has potentially free MREs both on mRNA targets and non-coding decoys, could be less prone to mimic activity. Therefore, future studies should consider the integrated evaluation of a multilayered regulatory network employing miRNA, long non-coding RNA and coding transcripts in order to envisage the potential mechanisms with translational potential for therapeutic application. As observed both in our and other studies, *MDM2* gene is regulated by multiple microRNAs associated with different pathologies [64–66]. Thus, we suggest that in future research a combination of miRNAs should be considered in order to achieve the maximum compensatory effect of specific miRNA deletion due to the potentially dysregulated ceRNA network.

The most intriguing results were observed in case of *NOTCH3* gene. NOTCH signaling has a significant role during nephrogenesis [35]. NOTCH3 was observed to be upregulated in dilated tubules and certain tubulointerstitial cells in unilateral ureteral obstruction (UUO) mice [36]. However, *NOTCH3* expression was significantly reduced in the HEK293 MIR484 KO^het^ cell line compared to HEK293 WT [10]. We have additionally observed that treatment of both HEK293 MIR484 KO^het^ and HEK293 WT cell lines using the hsa-miR-484 mimic, also leads to significant reduction of *NOTCH3* compared to NT miRNA control treatment (which is in line with the expected interaction between *NOTCH3* and hsa-miR-484). Thus the observed upregulation in HEK293 MIR484 KO^het^ compared to HEK293 WT cell line could be a phenomenon describing DNA lesion as a trigger for the transcriptional adaptation response, reviewed by El-Brolosy and colleagues [67] which could rescue the expected phenotype effect. These observations indicate that regulation can be unexpectedly dynamic. While the precise mechanism of the reduction of *NOTCH3* expression after transfection of hsa-miR-484 remains to be established, our results have potentially important impact for the use of hsa-miR-484 in stabilization of upregulated *NOTCH3* associated with CAKUT phenotypes.

## Conclusion

Our findings have demonstrated that restitution of hsa-miR-484 and hsa-miR-185-5p levels previously affected by CAKUT-associated CNVs has a very complex effect on downstream mRNA regulation. To overcome the limitations of the current study, the observed scenarios of a convoluted regulation of miRNA-gene expression should be further investigated in integrative studies involving both chromatin dynamics, compensatory effects and miRNA-mRNA-lncRNA interactions and on protein levels. A tissue-specific conditional activation/deactivation of miRNA genes in in vivo models will also provide future insights on the mechanism of CNV-affected miRNAs. Additionally, a correlation between miRNA CNV genotype and corresponding target gene expression should be performed ex vivo to evaluate the effect in the context of compensatory mechanisms. Furthermore, future studies should consider joint treatment with preassembled miRNA–Argonaute complexes [68] as it was described that the amount of free Argonaute could limit the functional effect of transfected miRNAs [69]. Dysregulation of multiple mRNA targets mediated by CNV-affected miRNAs could lead to CAKUT phenotype variability. Although this study should be considered as preliminary, the functional characterization of miRNAs located in CNVs which are expressed in a dose dependent manner could eventually lead to a better understanding of CAKUT and improving the management of patients.

### Electronic supplementary material

Below is the link to the electronic supplementary material.

Supplementary Material 1

## Data Availability

The datasets supporting the conclusions of this article are included within the article (and its Additional File).

## Abbreviations

APAF1: Apoptosis protease-activating factor-1
ATF6: Activating transcription factor 6
CAKUT: Congenital anomalies of the kidney and urinary tract
CDC42: Cell division cycle 42
CNVs: Copy Number Variants
DMEM: Dulbecco’s modified Eagle’s medium
FBS: fetal bovine serum
FIS1: Mitochondrial Fission 1 Protein
GAPDH: Glyceraldehyde 3-phosphate dehydrogenase
MDM2: Mouse double minute 2 homolog
MREs: miRNA response elements
NOTCH3: Neurogenic locus notch homolog
PKD1: Polycystin-1
RHOA: Ras homolog family member A
UUO: Unilateral Ureteral Obstruction
