# Supplementary material for: A preliminary study of the miRNA restitution effect on CNV-induced miRNA downregulation in CAKUT
Source: BMC Genomics. 2024 Feb 27;25:218. doi: 10.1186/s12864-024-10121-8 (PMC10900603; doi:10.1186/s12864-024-10121-8)
Supplement: Supplementary file 1 — Supplementary Material 1 [file 12864_2024_10121_MOESM1_ESM.pdf]

Additional File

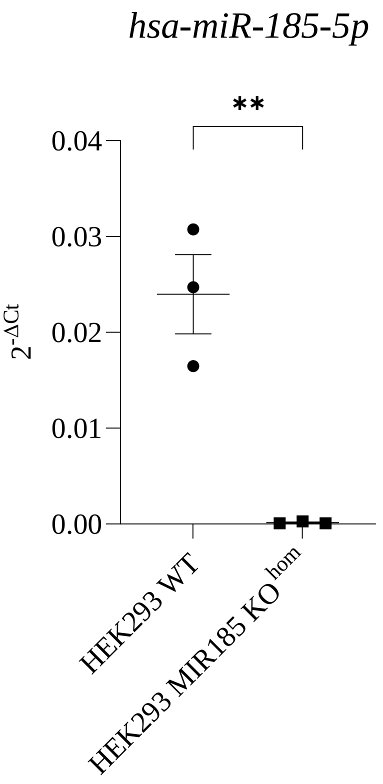

Figure A1. Difference in relative expression levels of hsa-miR-185-5p between HEK293 MIR185 KO^{hom} and HEK293 WT cells. Relative miRNA levels were standardized against RNU44 endogenous control and presented as a scatter plot of 2^{-dCt} values with standard errors of mean from three independent replicates. Student's t-test, P= 0,0045. ** denotes a significant difference at $P < 0.01$.

Table A1. Raw Ct values of *RNU44* and *hsa-miR-185-5p* after treatment of HEK293 MIR185 KO^{hom} cell line with 50nM of hsa-miR-185-5p mimic.

	Ct values	
**Sample**	**RNU-44**	***hsa-miR-185-5p***
NT miRNA control 1	27,138	Undetected
NT miRNA control 2	26,115	37,861
NT miRNA control 3	26,411	Undetected
hsa-miR-185 mimic 1	26,327	20,84
hsa-miR-185 mimic 2	26,21	21,92
hsa-miR-185 mimic 3	25,861	19,153

Based on uniform Ct values of endogenous control (*RNU44*) and detected differences in Ct values of *hsa-miR-185-5p* between hsa-miR-185-5p mimic and NT miRNA control transfected HEK293 MIR185 KO^{hom} cells, successful transfection of *hsa-miR-185-5p* mimic was confirmed. Lower Ct value corresponds with higher level of miRNA.

Table A2. Raw Ct values of *RNU44* and *hsa-miR-185-5p* after treatment of HEK293 WT cell line with 50nM of hsa-miR-185-5p mimic.

	Ct values	
Sample	**RNU44**	***hsa-miR-185-5p***
NT miRNA control 1	25,613	32,083
NT miRNA control 2	25,363	32,207
NT miRNA control 3	25,467	31,734
hsa-miR-185 mimic 1	25,171	18,771
hsa-miR-185 mimic 2	25,202	18,934
hsa-miR-185 mimic 3	25,454	18,973

Based on uniform Ct values of endogenous control (*RNU44*) and detected differences in Ct values of *hsa-miR-185-5p* between hsa-miR-185-5p mimic and NT miRNA control transfected HEK293 WT cells, successful transfection of *hsa-miR-185-5p* mimic was confirmed. Lower Ct value corresponds with higher level of miRNA.

Table A3. Raw Ct values of *RNU44* and *hsa-miR-484* after treatment of HEK293 MIR484 KO^{het} cell line with 50nM of hsa-miR-484 mimic.

	**Ct values**	
**Sample**	**RNU44**	***hsa-miR-484***
NT miRNA control 1	26,371	28,067
NT miRNA control 2	25,881	27,443
NT miRNA control 3	26,36	27,624
hsa-miR-484 mimic 1	26,28	16,386
hsa-miR-484 mimic 2	25,754	16,571
hsa-miR-484 mimic 3	25,915	16,862

Based on uniform Ct values of endogenous control (*RNU44*) and detected differences in Ct values of *hsa-miR-484* between hsa-miR-484 mimic and NT miRNA control transfected HEK293 MIR484 KO^{het} cells, successful transfection of *hsa-miR-484* mimic was confirmed. Lower Ct value corresponds with higher level of miRNA.

Table A4. Raw Ct values of *RNU44* and *hsa-miR-484* after treatment of HEK293 WT cell line with 50nM of hsa-miR-484 mimic.

	**Ct values**	
Sample	**RNU44**	***hsa-miR-484***
NT miRNA control 1	25,613	25,308
NT miRNA control 2	25,363	25,158
NT miRNA control 3	25,467	24,934
hsa-miR-484 mimic 1	25,484	15,458
hsa-miR-484 mimic 2	25,337	15,037
hsa-miR-484 mimic 3	25,669	15,194

Based on uniform Ct values of endogenous control (*RNU44*) and detected differences in Ct values of *hsa-miR-484* between hsa-miR-484 mimic and NT miRNA control transfected HEK293 WT cells, successful transfection of *hsa-miR-484* mimic was confirmed. Lower Ct value corresponds with higher level of miRNA.
